# Kikuchi-Fujimoto lymphadenitis imitating metastatic melanoma on positron emission tomography: a case report

**DOI:** 10.1186/s12893-015-0036-y

**Published:** 2015-04-28

**Authors:** Peter Urbanellis, Laura Chin-Lenn, Carolin J Teman, J Gregory McKinnon

**Affiliations:** Queen’s University School of Medicine, Kingston, Ontario Canada; Department of Surgical Oncology, Tom Baker Cancer Centre, University of Calgary, Calgary, Canada; Department of Pathology, Divisions of Anatomical Pathology and Cytopathology, and Hematopathology and Transfusion Medicine, University of Calgary, Calgary, Canada

**Keywords:** Kikuchi-Fujimoto disease, Positron emission tomography-computed tomography, Metastatic melanoma

## Abstract

**Background:**

Accurate staging is critical for decision-making for the treatment of malignant conditions. Fluoro-deoxy-glucose positron emission tomography-computed tomography (FDG PET-CT) is a highly sensitive imaging modality for the assessment of distant metastases; however false positive results are possible due to its lower specificity with detection of other hypermetabolic pathologies.

**Case presentation:**

A patient with high-risk thigh melanoma was staged with FDG PET-CT. Four ipsilateral inguinal nodes (three superficial, one deep) demonstrated intense hypermetabolic activity. Metastatic melanoma was confirmed in the largest superficial inguinal node with ultrasound-guided fine needle aspiration. Histopathology demonstrated metastatic melanoma in one superficial node and histiocytic necrotizing lymphadenitis, also known as Kikuchi-Fujimoto disease in five deep inguinal nodes.

**Conclusion:**

This case illustrates a false positive FDG PET-CT due to coincidental, synchronous melanoma and Kikuchi-Fujimoto disease in the same draining lymph node basin.

## Background

Kikuchi-Fujimoto disease (KFD) is a rare, benign condition of unknown etiology that mostly affects young women. It typically manifests as cervical lymphadenopathy and uncommonly as widespread lymphadenopathy, and is associated with constitutional symptoms of fever, malaise, and weight loss. Affected lymph nodes may display increased avidity on fluoro-deoxy-glucose positron emission tomography (FDG PET) and on histopathology show a mitotically active lymphoid infiltrate with necrosis, sometimes leading to a misdiagnosis of malignancy [1]. Here, we describe a woman with melanoma of the leg with FDG-avid superficial and deep inguinal lymph nodes, found to be due to concurrent metastatic melanoma and KFD.

## Case presentation

A 37 year old Caucasian woman presented to our referral centre with invasive melanoma. For one year she had noticed a posterior thigh raised pigmented lesion, which rapidly grew over three months. It measured 2 cm and bled spontaneously. She had no constitutional symptoms such as fever, malaise, or weight loss, or significant medical history. Histopathology of the initial shave biopsy demonstrated invasive melanoma with Breslow thickness greater than 4.9 mm (positive deep margin), Clark level IV, ulceration, and a mitotic rate of 28 mitoses per square millimeter.

Physical examination revealed a 2.5 cm healing biopsy site on the left thigh with residual pigmentation. Further examination was unremarkable, with no satellite lesions, in-transit disease, palpable lymphadenopathy or abdominal organomegaly. No additional suspicious skin lesions were identified.

**Figure 1:**
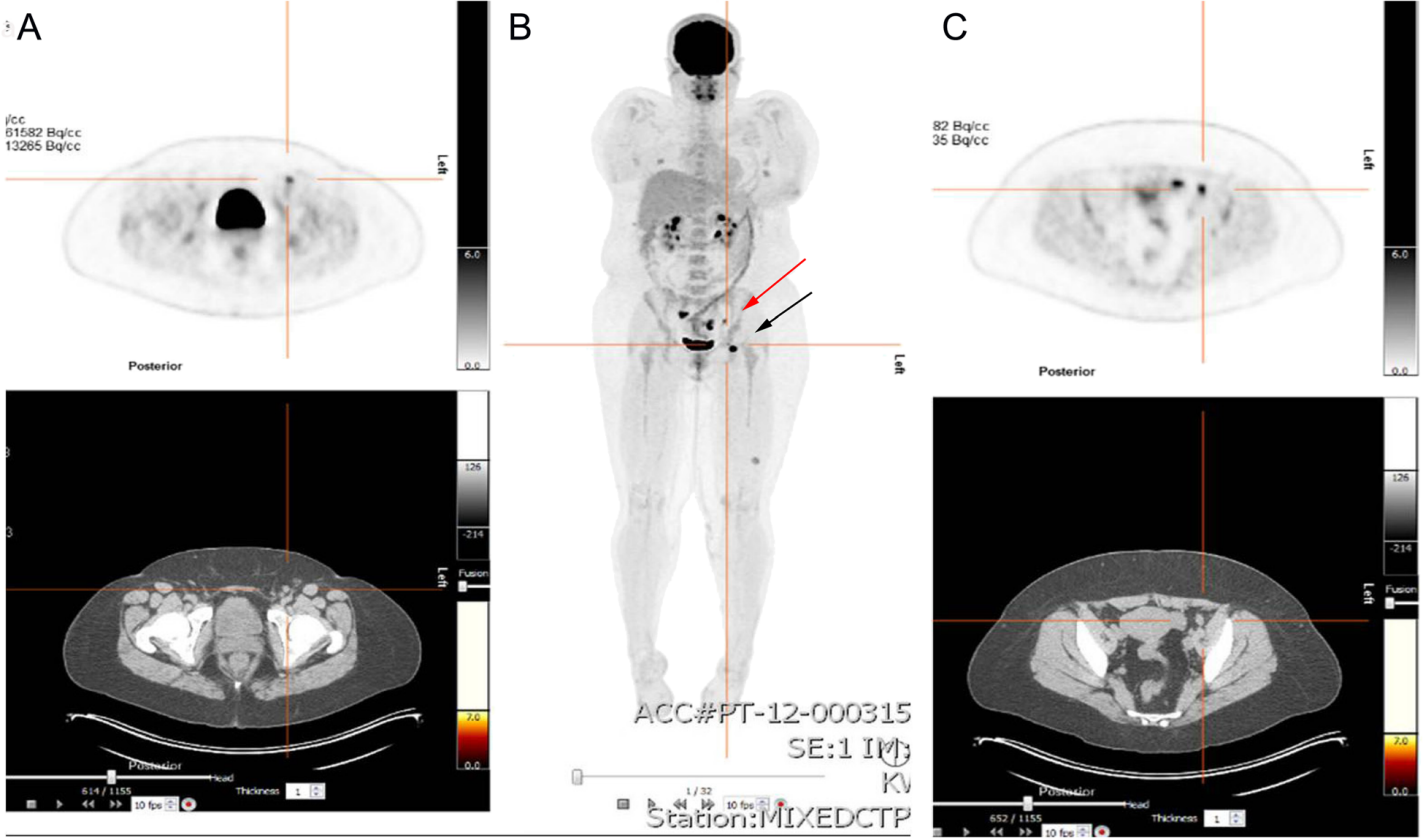
FDG PET and CT images **(A)** Axial FDG PET and CT images demonstrating intense avidity in a left superficial inguinal lymph node; **(B)** Whole body FDG PET demonstrating intense avidity in three inguinal superficial lymph nodes (black arrow) and left deep inguinal (external iliac) lymph node (red arrow). Hypermetabolism is seen in the left thigh biopsy site and likely physiologic changes in the right and left adnexae. **(C)** Axial FDG PET and CT images demonstrating the avid left deep inguinal lymph node.

Due to the aggressive pathological features of the primary lesion and difficulty with clinical nodal assessment due to the patient’s body habitus, she was staged with FDG PET-CT. Intense hypermetabolic activity was seen in the ipsilateral left superficial inguinal lymph node basin, the largest of which measured 19 x 14 mm, and two or three adjacent tiny lymph nodes. Additionally, a single 9 mm deep inguinal (external iliac) lymph node demonstrated intense hypermetabolic activity (Figure 1A and B). Moderate hypermetabolic activity was seen on the posterior left thigh corresponding to the residual neoplasm and biopsy site. There was some physiological FDG uptake but no further suspicious areas identified. Ultrasound-guided fine-needle aspiration of the largest superficial left inguinal lymph node demonstrated metastatic melanoma.

The patient underwent a wide local excision of the primary tumor and left superficial and deep inguinal node dissection.

**Figure 2:**
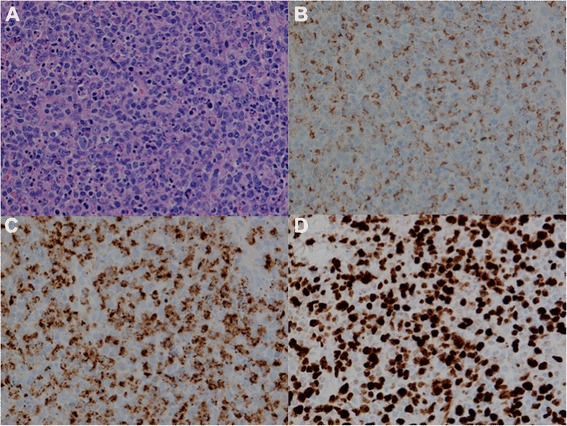
Kikuchi-Fujimoto disease histopathology **A** Sheet-like lymphohistiocytic infiltrate with crescentic histiocytes and abundant karyorrhectic debris (hematoxylin-eosin, 400x magnification). **B** Histiocytes are highlighted by CD68 (400x magnification). **C** Histiocytes co-express myeloperoxidase (400x magnification). **D** High proliferative index is highlighted by Ki-67 (400x magnification).

Pathology identified eleven lymph nodes in the superficial and deep lymphadenectomy specimen. One of the superficial left inguinal lymph nodes contained metastatic melanoma, confirmed by HMB-45 and melan-A positivity. However, five deep inguinal lymph nodes (2 large and 3 smaller nodes) were partially involved by a florid atypical lymphohistiocytic proliferation with a brisk mitotic rate and abundant necrosis. The specimen was referred for Hematopathology consultation due to a concern of high-grade lymphoma. The involved lymph nodes showed partial architectural effacement by a mixed proliferation of small lymphocytes, large immunoblast-like lymphocytes, and histiocytes, present in a predominantly paracortical distribution. Mitotic rate in these areas was very brisk (up to 10 mitoses per high power field). Many of the histiocytes had eccentric crescent-shaped nuclei and contained abundant intracytoplasmic karyorrhectic debris (Figure 2A). Neutrophils and eosinophils were noticeably absent. In some areas, the cellular proliferation was entirely replaced by large zones of necrosis. Fibrin thrombi were identified within numerous blood vessels in and around the necrotic areas. By immunohistochemistry, most of the lymphocytes were CD8 positive cytotoxic T-cells. The abundant histiocytes were highlighted by CD68 (Figure 2B) and lysozyme, and also showed strong expression of myeloperoxidase (Figure 2C). Ki-67 staining confirmed a high proliferative index of approximately 80% (Figure 2D). The uninvolved areas of the lymph node showed reactive follicular hyperplasia and fine septal fibrosis, but were otherwise normal. These morphologic and immunophenotypic features are classic for histiocytic necrotizing lymphadenitis, also known as KFD. This proliferation was morphologically and immunophenotypically distinct from the patient’s concurrent metastatic melanoma, which consisted of sheets of large, pleomorphic epithelioid cells with expression of HMB-45 and melan-A.

## Discussion

KFD is a poorly understood and rare condition which most commonly affects young women of Asian descent, but also males and children. The true incidence of KFD is difficult to ascertain due to its heterogeneous, often subtle symptoms. KFD typically presents as tender cervical lymphadenopathy, but may involve other lymph node regions and rarely presents as generalized lymphadenopathy. While it is often otherwise asymptomatic, KFD may also exhibit a constellation of non-specific symptoms including fever, upper respiratory tract symptoms, weight loss, night sweats, fatigue, nausea and vomiting [1]. Uncommonly there may be cutaneous manifestations such as rash, macules or papules, or ulcers [2]. KFD usually develops over two to three weeks and spontaneously resolves after a few months. Treatment is generally supportive care only but severe cases may require non-steroidal anti-inflammatories or corticosteroids. Hydroxychloroquine, methotrexate, and intravenous immunoglobulin have also been used for severe cases [2]. KFD may relapse and exceptionally rare cases have resulted in death [3].

Imaging findings are usually non-specific, and KFD may be mistaken for lymphoma, other malignancies, tuberculosis lymphadenitis, or Kawasaki disease [1]. The number, size (usually <4 cm) and location (neck most common) of involved lymph nodes can be useful for suggesting the diagnosis [4]. Because of the brisk proliferation rate, lymph nodes involved by KFD are hypermetabolic on FDG PET-CT. The maximum standard uptake values (SUVmax) of FDG uptake can be used to differentiate benign and malignant tumors, but in a series of seven patients with KFD, the SUVmax ranged from 2.05-13.94 (mean +/−SD, 6.25+/−3.32), which is similar to malignancies [4]. In that study, lymph node size weakly correlated with SUVmax, but the authors suggested that the FDG uptake was more dependent on the number of macrophages and neutrophils in the lymph node tissue [4]. The diagnosis is most reliably made by histopathologic examination of an excised affected lymph node.

The etiology of KFD remains highly speculative. The clinical symptoms and pathological findings suggest a viral cause but no specific agent has been identified. It has been proposed that an infectious process initiates a self-limited autoimmune response. However, reports have been conflicting, with some implicating viruses such as Epstein-Barr virus, human herpes virus 6, 7, 8 and parvovirus B19, but others refuting them [5,6]. It is still possible that KFD develops after eradication of these viruses, or that other viruses contribute to the pathology [6]. KFD has also been associated with autoimmune conditions, in particular systemic lupus erythematosus (SLE). The association is not well defined, and it is unclear if SLE can present with lymphadenopathy with similar pathological features of KFD or if KFD may be an early manifestation of SLE. Nevertheless, this correlation has led some physicians to recommend following KFD patients clinically for SLE [2].

To our knowledge, there are no other reported cases of a simultaneous diagnosis of melanoma and KFD. Melanoma is susceptible to immune modulation. Although there has been an association noted between SLE and melanoma, a large Danish population-based study found that the incidence of melanoma was not increased in those with autoimmune diseases [7,8]. In our patient, the only FDG-avid lymphadenopathy was in the regional lymph node basin draining the melanoma. It is unclear whether this was due to a possible immunological association between melanoma and KFD, whether there was a reaction from the biopsy of the primary site or if whether the co-existence is pure coincidence.

NCCN guidelines recommend staging with FDG PET or CT for stage III and IV melanoma [9]. In this case, a FDG PET-CT was requested because of the unusually aggressive features of the primary tumor, as well as the patient’s body habitus that made clinical examination of lymph nodes difficult. Potential drawbacks in the use of FDG PET-CT or CT for staging are the possibility of false positive results that vary in incidence between 0-27%, which are more common when staging nodal disease compared with visceral metastases [10].

In our patient, a superficial inguinal lymphadenectomy was appropriately performed for fine-needle aspiration proven metastatic melanoma. However, evidence of deep lymph node involvement on FDG PET-CT also prompted a deep inguinal lymphadenectomy. Without concomitant KFD, only superficial nodal involvement would have been identified on FDG PET-CT and a superficial inguinal lymph node dissection would have been considered adequate.

## Conclusions

In summary, we report a patient with synchronous metastatic melanoma and KFD found during inguinal lymphadenectomy. This case highlights a possible association between the two diseases and a potential pitfall of false-positive FDG-PET imaging. FDG PET-CT findings initially indicated hypermetabolic nodes in the superficial and deep inguinal lymph nodes that were suspicious for metastatic spread of melanoma given the patient’s clinical history. Although melanoma was identified in the superficial nodes, it was only after surgical removal and histological evaluation that KFD was identified in the deep inguinal chains. This case illustrates the importance of recognizing the possible false positives found with FDG PET-CT due to concurrent hypermetabolic pathology.

### Consent

Written consent was obtained from the family for publication of this case report and accompanying images as the patient is deceased. A copy of the written consent is available for review by the Editor of this journal.

## Abbreviations

CD: Cluster of differentiation
Cm: Centimetre
FNA: Fine needle aspiration
HMB-45: Human Melanoma Black 45
KFD: Kikuchi-Fujimoto Disease
Mm: Millimetre
FDG: Fluoro-deoxy-glucose
PET: Positron emission tomography
PET-CT: Positron emission tomography-computed tomography
SLE: Systemic Lupus Erythematosus
